# Novel Marmoset (*Callithrix jacchus*) Model of Human Herpesvirus 6A and 6B Infections: Immunologic, Virologic and Radiologic Characterization

**DOI:** 10.1371/journal.ppat.1003138

**Published:** 2013-01-31

**Authors:** Emily Leibovitch, Jillian E. Wohler, Sheila M. Cummings Macri, Kelsey Motanic, Erin Harberts, María I. Gaitán, Pietro Maggi, Mary Ellis, Susan Westmoreland, Afonso Silva, Daniel S. Reich, Steven Jacobson

**Affiliations:** 1 Viral Immunology Section, Neuroimmunology Branch, NINDS/NIH, Bethesda, Maryland, United States of America; 2 Institute for Biomedical Sciences, George Washington University, Washington, DC, United States of America; 3 New England Primate Research Center, Harvard Medical School, Southborough, Massachusetts, United States of America; 4 Translational Neuroradiology Unit, Neuroimmunology Branch, NINDS/NIH, Bethesda, Maryland, United States of America; 5 Cerebral Microcirculation Unit, Laboratory of Functional and Molecular Imaging, NINDS/NIH, Bethesda, Maryland, United States of America; University of North Carolina, United States of America

## Abstract

Human Herpesvirus 6 (HHV-6) is a ubiquitous virus with an estimated seroprevalence of 95% in the adult population. HHV-6 is associated with several neurologic disorders, including multiple sclerosis, an inflammatory demyelinating disease affecting the CNS. Animal models of HHV-6 infection would help clarify its role in human disease but have been slow to develop because rodents lack CD46, the receptor for cellular entry. Therefore, we investigated the effects of HHV-6 infections in a non-human primate, the common marmoset *Callithrix jacchus*. We inoculated a total of 12 marmosets with HHV-6A and HHV-6B intravenously and HHV-6A intranasally. Animals were monitored for 25 weeks post-inoculation clinically, immunologically and by MRI. Marmosets inoculated with HHV-6A intravenously exhibited neurologic symptoms and generated virus-specific antibody responses, while those inoculated intravenously with HHV-6B were asymptomatic and generated comparatively lower antibody responses. Viral DNA was detected at a low frequency in paraffin-embedded CNS tissue of a subset of marmosets inoculated with HHV-6A and HHV-6B intravenously. When different routes of HHV-6A inoculation were compared, intravenous inoculation resulted in virus-specific antibody responses and infrequent detection of viral DNA in the periphery, while intranasal inoculation resulted in negligible virus-specific antibody responses and frequent detection of viral DNA in the periphery. Moreover, marmosets inoculated with HHV-6A intravenously exhibited neurologic symptoms, while marmosets inoculated with HHV-6A intranasally were asymptomatic. We demonstrate that a marmoset model of HHV-6 infection can serve to further define the contribution of this ubiquitous virus to human neurologic disorders.

## Author Summary

The human herpesviruses HHV-6A and HHV-6B are widely distributed in the human population, but also specifically associated with several central nervous system (CNS) diseases. We investigated HHV-6A and HHV-6B infections in the common marmoset, a non-human primate naturally susceptible to infection, unlike rodents. We inoculated marmosets with HHV-6A and HHV-6B intravenously, and with HHV-6A intranasally, to represent a more physiologic route of infection. Following intravenous HHV-6A inoculation, marmosets exhibited clinical symptoms with evidence of spinal cord pathology. Animals inoculated intravenously with HHV-6B were asymptomatic and without detectable CNS pathology. Both groups developed robust anti-viral antibody responses, and we detected viral DNA infrequently in the periphery. By contrast, marmosets inoculated intranasally with HHV-6A were asymptomatic, failed to generate anti-viral antibodies, and we frequently detected viral DNA in the periphery. Interestingly, HHV-6 DNA was detected in brain and spinal cord sections of several intravenously inoculated animals, demonstrating that HHV-6 can gain access to and persist in the CNS. These observations help to define the contributions of ubiquitous herpesviruses to neurologic disease development in a non-human primate. As little is known about the acquisition and host response to HHV-6A, this model may clarify how this virus may trigger or potentiate disease.

## Introduction

Human Herpes Virus 6 (HHV-6) is a member of the *Roseolovirus* genus of the β-herpesvirus subfamily [1]. Since its identification in 1986, two species, HHV-6A and HHV-6B, have been described [2]. Though HHV-6A and HHV-6B share high sequence homology, they differ in cellular tropism and clinical manifestation [3], [4], [5], [6] to the extent that they were recently reclassified as two distinct viruses (International Committee on Taxonomy of Viruses, 2011). Primary infection with HHV-6B is often associated with febrile illness [7], and this virus is the etiologic agent of the self-limiting childhood illness roseola infantum [8]. By contrast, the symptoms associated with HHV-6A infection are largely unknown.

HHV-6 is acquired during early childhood [7]. The virus has a worldwide distribution, with an estimated seroprevalence of 95% in the adult population [9], [10]. HHV-6 cell tropism is notably lymphotropic and neurotropic, though it can infect a wide range of human cells *in vitro* due to the ubiquity of its major receptor, CD46 [11]. Similar to other herpesviruses, HHV-6 can establish lifelong latent, asymptomatic infections [12]. However, the virus may reactivate as a consequence of immunosuppression, manifesting for example as a febrile illness [13] or encephalitis following bone marrow [14] or solid organ [15] transplantation.

HHV-6 DNA has been reported in normal brain tissues [16] suggesting that this virus may be a commensal of the brain under some circumstances [17]. However, HHV-6 is also associated with neurologic conditions including encephalitis [18] [19] [20], temporal lobe epilepsy [21] [22] and multiple sclerosis (MS) [23], [24], [25], findings that have been established by assessing both the distribution of viral DNA and serologic responses. HHV-6 DNA is found in MS lesions [26] [27] [28]. Moreover, HHV-6 DNA has been detected in cell-free compartments, such as the sera and urine, of some MS patients [29], and is detected at higher frequencies during periods of clinical exacerbation relative to periods of remission. As HHV-6 is normally cell-associated, the detection of viral DNA in cell-free compartments suggests an active infection [30]. More recently, significantly elevated serum HHV-6 IgM in MS patients versus controls was reported in an Iranian population [31], and a positive, dose-dependent correlation of serum HHV-6 IgG titers with MS relapse risk was reported in an Australian MS cohort [32].

Despite the association of HHV-6 with several central nervous system (CNS) disorders [33], [34], [19] it has been difficult to prove causation in clinical disease. This is partly due to the ubiquity of HHV-6 infection in the general population and also because no animal model exists. Animal models of HHV-6 infection have been difficult to establish because rodents lack the complement regulatory receptor, CD46, that HHV-6 uses for cellular entry [35]. The common marmoset (*C. jacchus*) is a New World non-human primate that naturally expresses CD46 [36] and is therefore susceptible to infection with HHV-6.

Marmoset models of various neurologic diseases have been developed [37], including the animal model for MS, experimental autoimmune encephalomyelitis (EAE). EAE is an inflammatory, demyelinating disease of the CNS induced by immunization with myelin antigen(s) [38], [39], [40], [41]. It is increasingly apparent that marmoset EAE (relative to rodent EAE) has superior translational applicability, due to greater similarities with MS such as CD8 T cell involvement, the presence of both brain and spinal cord lesions and importantly, the ability for MRI analysis of lesions [39]. Marmosets are particularly appropriate for studies involving MRI monitoring because the cerebral organization resembles, but is considerably simpler than that of humans. Moreover, these primates are ideal models for studying the pathogenesis and host response to a human virus in a non-human system due to their genetic and immunologic proximity to humans, in addition to their broad behavioral range [39]. Marmosets have been infected with other human herpesviruses, such as Varicella Zoster virus (VZV) [42], Kaposi's sarcoma-associated herpesvirus (KSHV) [43], as well as non-herpesviruses such as dengue virus (DENV) [44].

In this study, marmosets were inoculated with HHV-6A or HHV-6B intravenously, or with HHV-6A intranasally. Intranasal inoculation was examined based on a recent report demonstrating the olfactory pathway as a possible route of HHV-6 entry into the CNS [45]. All resulting infections were monitored clinically, immunologically and by MRI for 25 weeks following the first inoculation. Previous work has demonstrated that following intravenously administered HHV-6-infected cell lysates, marmosets can develop hypotonic paralysis with sensory deficits accompanied by weight loss (Genain, C., unpublished data, 6^th^ international conference on HHV-6 & 7). Here we report that marmosets inoculated intravenously with HHV-6A exhibit neurologic symptoms, mount virus-specific IgM and IgG responses and effectively clear the virus from peripheral circulation. By contrast, marmosets inoculated intranasally with HHV-6A are asymptomatic, do not mount virus-specific IgM or IgG responses and fail to clear the virus from peripheral circulation within the 25-week monitoring period of this study. These observations suggest that the route of inoculation is an important determinant for establishing humoral immunity, and that humoral immunity may influence not only the peripheral circulation of viral DNA, but also pathological features such as clinical symptoms and CNS pathology.

## Methods

### Animals

Fifteen adult common marmosets (*Callithrix jacchus*) (Table 1) were used in this study. Marmosets were singly housed with a twelve-hour light/dark cycle on a diet of Zupreem canned marmoset food, Purina 5040 biscuits, fruit and vegetable treats and *ad libitum* unfiltered water and PRANG rehydrator.

**Table 1:** Marmoset demographics and summary of results by experimental group.

Experimental group	Marmoset	Sex/Age at study start (months)	Time to clinical symptoms (days)	Time between viral inoculation and sacrifice (months)	CNS samples positive for HHV-6 DNA/total surveyed	CNS region(s) positive for HHV-6 DNA
**HHV-6A intravenous**	M01*	F/173	49	14	0/9	
	M02*	F/45	29	12	0/10	
	M03*	M/109	64	14	0/10	
	M04*	M/28	27	14	1/9	• Occipital cortex/cerebellum
**HHV-6B intravenous**	M05*	M/99	–	8.5	1/10	• Occipital cortex/cerebellum/brain stem
	M06	M/147	–			
	M07*	F/47	–	9	4/10	• Occipital cortex/cerebellum
						• Prefrontal cortex
						• Basal ganglia
						• Cervical/thoracic spinal cord
	M08*	F/42	–	16	0/8	
**Vehicle Control intravenous**	M09	F/29	–			
	M10	F/29	–			
	M11	F/20	–			
**HHV-6A intranasal**	M12*	M/55	–	6.5	0/11	
	M13	F/66	–			
	M14	F/53	–			
	M15	M/47	–			
					**Total: 6/77 (8%)**	

### Ethics statement

All marmosets were housed at the National Institutes of Health Intramural Research Program (PHS Assurance #A4149-01) facilities in accordance with the standards of the American Association for Accreditation of Laboratory Animal Care and the National Institute of Neurological Disorders and Stroke's Internal Animal Care and Use Committee (NINDS IACUC). All experiments adhered to a protocol that was reviewed and approved by the NINDS IACUC.

### Virus and infection

HHV-6A (U1102) and HHV-6B (Z29) were separately propagated in the T-lymphoblastoid cell line SupT1 as described previously [46]. The supernatants of infected cells were quantified using real time PCR, with primers to detect the intermediate early U90 region of the HHV-6 genome as described previously [47]. Supernatants were stored at −80°C until use. Marmosets were anesthetized with ketamine (10 mg/kg) prior to viral inoculations. Three groups of marmosets were injected intravenously with HHV-6A supernatants (1×10^9^ viral copies of DNA) (n = 4; M01–M04), HHV-6B supernatants (1×10^9^ viral copies of DNA) (n = 4; M05–M08) or mock-infected supernatants from uninfected SupT1 cells (n = 3; M09–M11) (Table 1). Marmosets were re-exposed intravenously once a month for a total of four doses (4×10^9^ total viral copies of DNA). A fourth group of marmosets was induced with HHV-6A supernatant intranasally (2×10^7^ viral copies of DNA) (n = 4; M12–M15) (Table 1). Marmosets were re-exposed intranasally once a month for a total of three doses (6×10^7^ total viral copies of DNA).

### Disease assessment

Following HHV-6 inoculation, all marmosets were monitored and scored daily for signs of disease development. Clinical signs were scored using a previously described semiquantitative scale commonly used to assess marmoset EAE [48]. Briefly, 0: no clinical signs; 0.5: apathy or altered walking pattern without ataxia; 1: lethargy or tremor; 2: ataxia or optic disease; 2.25: monoparesis; 2.5: paraparesis or sensory loss; 3: paraplegia or hemiplegia. Body weights were measured three times per week and prior to each MRI, marmosets were subject to a neurologic exam performed by a neurologist.

### Sample collection and DNA extraction

Marmosets were anesthetized with ketamine (10 mg/kg) intramuscularly prior to blood sampling. Approximately 1 cc blood was drawn from the femoral triangle of each animal prior to HHV-6 inoculation and every two weeks post-inoculation. PBMC were isolated using Lymphocyte Separation Medium (Mediatech, VA) and plasma was collected from the isolation. Saliva was collected at the time of blood sampling using gauze to swab the mouth of the animal. Saliva was diluted with PBS and spun out of the gauze for DNA extraction. DNA extraction from PBMC, plasma, saliva and organs collected at euthanasia was performed with the DNeasy Blood and Tissue DNA extraction kit (Qiagen, CA). DNA extraction from 10 µM scrolls from paraffin-embedded brain and spinal cord sections was performed with the QIAamp DNA formalin fixed paraffin embedded (FFPE) tissue kit (Qiagen, CA).

### HHV-6-specific PCR

Nested PCR (nPCR) was used to monitor the presence of HHV-6 DNA in the plasma, PBMC and saliva every two weeks, as well as in the organs and paraffin-embedded sections following necropsy. HHV-6 nPCR was performed with primers against the U57 region (major capsid protein, MCP) of the viral genome, as described previously [20]. As this method does not distinguish between HHV-6A and HHV-6B, sequencing was conducted at the NINDS DNA sequencing facility (Bethesda, MD) to determine the species present in the PCR positive samples. All reactions were performed in triplicate, and PCR positive was defined as a positive result two out of three times. All extracted samples were tested for the presence of amplifiable DNA through the amplification of β-actin using real-time PCR [47]. For the paraffin-embedded CNS tissue, one 10 µM scroll was isolated from each section for DNA extraction. Only samples with amplifiable DNA, as defined by β-actin Ct values ≤35, were further analyzed for the presence of HHV-6 DNA (>97% of all samples tested).

### Detection of anti-HHV-6 IgG and IgM

Plasma antibodies against HHV-6 proteins were measured every two weeks for 25 weeks post-inoculation using electrochemiluminescence technology (MSD, Gaithersburg, MD) developed in our laboratory [49]. HHV-6A or mock-infected (SupT1) cell lysate, prepared as previously described [24], was spotted onto high bind plates and allowed to dry overnight at room temperature (RT). Plasma samples were diluted in MSD Antibody Diluent (final dilution 1∶10) and added to plates. Sulfo-Tag-labeled anti-human IgG (Jackson ImmunoResearch) was used to detect IgG responses and Sulfo-Tag-labeled polyclonal anti-human IgM (MSD) was used to detect IgM responses. Each sample was tested in duplicate, and signal intensity is expressed as light emitting units. Results are corrected for responses to uninfected SupT1 lysates and reported as fold increases over baseline (before viral inoculation).

### *In vivo* magnetic resonance imaging (MRI)

MRI scans of the brain were performed monthly following viral inoculation, and scans obtained during the experimental monitoring period were compared to baseline scans (conducted before viral inoculation). Before each MRI experiment, marmosets were fasted for 12 h, sedated with an intramuscular injection of 10 mg/kg ketamine and orally intubated. Throughout the imaging session, sedated marmosets were mechanically ventilated with a mixture of oxygen and 1.25–2% isoflurane, and physiological parameters including end-tidal CO_2_, heart rate, and SPO_2_ were monitored using a capnograph and pulse oximeter (Surgivet, Waukesha, WI, USA). Rectal temperature was also monitored, and maintained at 38.5°C with a water heating pad.

MRI was performed on a 7 T/30 cm USR/AVIII MRI scanner (BrukerBiospin Corp., Ettlingen, Germany) equipped with a 15 cm gradient set of 450 mT/m strength (Resonance Research Inc., Billerica, MA, USA). A custom-built, 16-rung, high-pass birdcage radiofrequency coil with a 12 cm inner diameter was used for transmission and a custom-built five-element receive-only phased array equipped with preamplifiers was used for reception. For all marmosets, the MRI protocol included T2-weighted Turbo Spin Echo (T2w-TSE), T1-weighted Magnetization Prepared Rapid Acquisition Gradient Echo (T1w-MPRAGE) and T1-weighted Fast Low Angle Shot imaging (T1w-FLASH) performed before the injection of contrast agent. A tail vein was cannulated for the administration of a bolus of Gadolinium-Diethylene-Triamine Penta-acetic Acid (Gd-DTPA; Magnevist). Each marmoset received 0.3 mMol/kg of Gd-DTPA over three minutes. T1w-FLASH imaging was repeated approximately 20 minutes after the injection of Gd-DTPA.

### *Post-mortem* magnetic resonance imaging (MRI)

At necropsy, animals were transcardially perfused with cold 4% paraformaldehyde (PFA) and whole brain and spinal cord were collected. The brain was placed in 10% neutral buffered formalin (NBF), and the spinal cord was cut into superior and inferior sections and then placed in 4% PFA. Tissue sections were submerged in nonmagnetic oil (Fomblin), and postmortem MR images were recorded in the same magnet using a volume transmit-receive RF coil with 40 mm diameter (Bruker-Biospin). For all marmosets, the post-mortem brain MRI protocol included T2w-TSE and T2*-weighted Multi Gradient Echo imaging (T2*-MGE). The spinal cord post-mortem imaging protocol included T2* weighted Fast Low Angle Shot Imaging (T2*-FLASH).

### Immunohistochemistry (IHC) and histological staining of CNS tissue

All animals were necropsied within 1 hour of death. Brains and spinal cords were fixed in 10% NBF and 4% PFA, respectively, and subsequently embedded in paraffin, sectioned at 5 µm and stained using hematoxylin and eosin (HE). Two special stains, Bielschowski's method for neurofibrils and luxol fast blue (LFB) for myelin, were additionally performed on all sections.

Standard immunoperoxidase IHC for ionized calcium binding adapter molecule one (Iba-1), a macrophage and microglia-specific marker, was also performed. Sections of brain and spinal cord were deparaffinized, rehydrated, and blocked with 3% hydrogen peroxide in PBS. Iba-1 pretreatment involved microwaving for 20 minutes in 0.01 citrate buffer, followed by 20 minutes of cooling. Following pretreatment, an avidin-biotin block (Invitrogen Corporation, Frederick, MD, USA) and a Dako Protein block (10 minutes; Carpineria, CA, USA) were conducted on all sections. A wash of tris-buffered saline (TBS) followed each step.

Sections were incubated with Iba-1 (Wako Pure Chemical Industries, Ltd., Osaka, Japan; polyclonal) at a 1∶1000 dilution for thirty minutes at RT. Slides were then incubated with a secondary antibody, biotinylated goat anti-rabbit (Vector Laboratories, Burlingame, CA, USA) at a 1∶200 dilution for 30 minutes at RT, followed by 30 minutes incubation at RT with Vectastain ABC Elite (Vector Laboratories, Burlingame, CA, USA). Antigen-antibody complex formation was detected using diaminobenzidine (DAB; DakoCyomation, Carpinteria, CA, USA) and counterstained with Mayer's hematoxylin. Irrelevant, isotype-matched primary antibodies were used in place of the test antibody as negative controls. Positive control tissue consisted of rhesus macaque spleen.

## Results

### Intravenous inoculation of HHV-6A results in neurologic symptoms

In our initial experiment, eight marmosets were inoculated intravenously with HHV-6A (n = 4) or HHV-6B (n = 4). Each marmoset received four inoculations, and daily monitoring was conducted for 180 days from the first inoculation.

**Figure 1:**
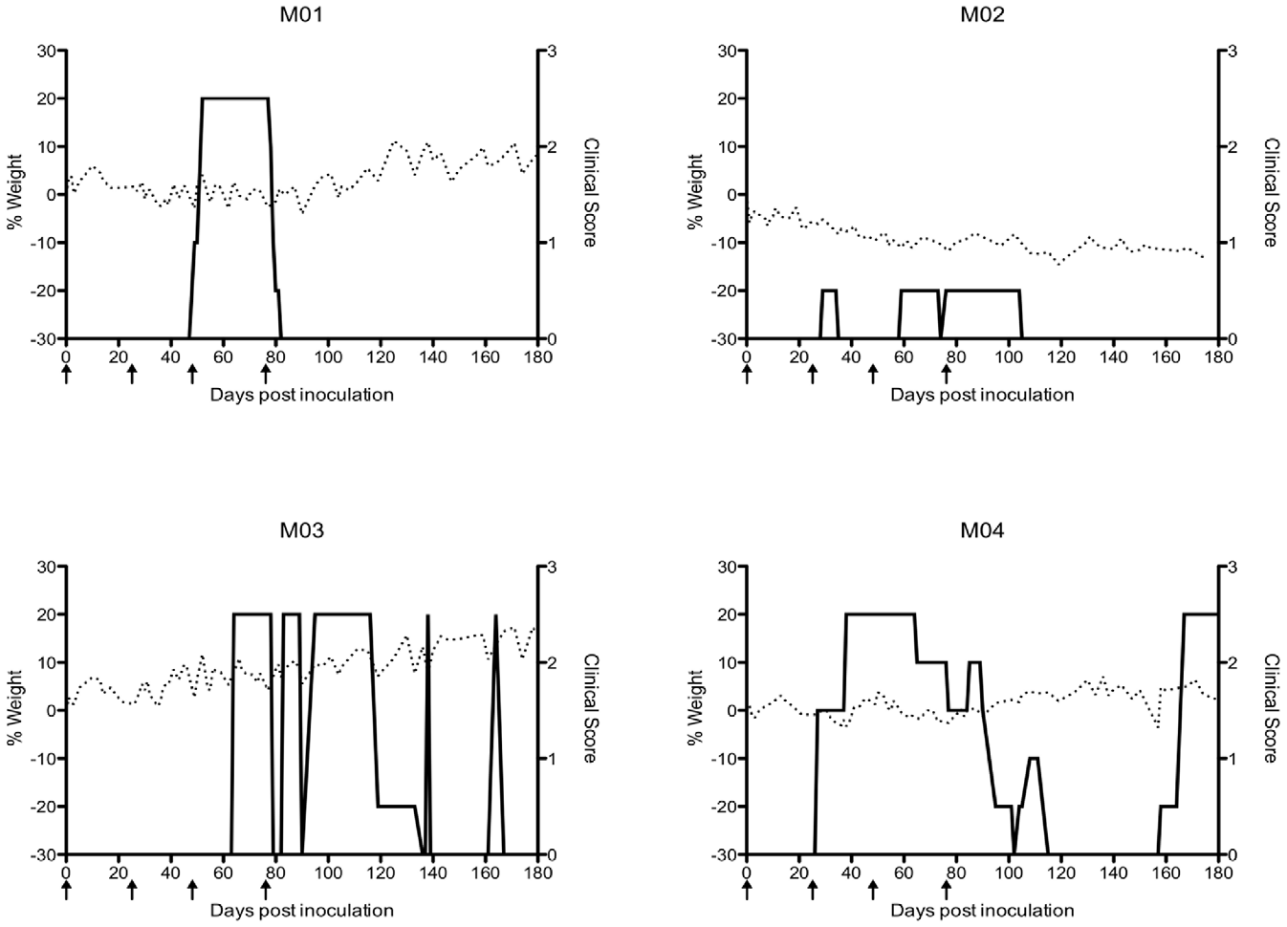
Marmosets inoculated intravenously with HHV-6A exhibited clinical symptoms without weight loss. Percent weight change is on the left y-axis (dashed line). Clinical score is on the right y-axis (solid line). The scoring system is as follows, 0: no clinical signs, 0.5: apathy or altered walking pattern without ataxia, 1: lethargy or tremor, 2: ataxia or optic disease, 2.25: monoparesis, 2.5: paraparesis or sensory loss, 3: paraplegia or hemiplegia. Arrows represent times of HHV-6A intravenous inoculations.

Shortly after the second intravenous inoculation of HHV-6A, neurologic symptoms developed in two of the four marmosets, M01 and M04 (Figure 1, solid lines). M01 presented with sensory and motor impairment in her left arm, characterized by flapping, which persisted for 29 days and then resolved (clinical score: 2.5). M04 first presented with a facial palsy characterized by a droopy lower lip and an inability to blink the eye on the affected side, which persisted for seven days and then resolved (clinical score: 1.5). M04 then presented with motor impairment in his left leg, holding it in retraction while moving. This lasted for 63 days and then resolved, but a recurrence of weakness in this leg was noted on day 167 post-inoculation and persisted through the end of the monitoring period (clinical score: 2.5).

Following the third intravenous inoculation of HHV-6A, M03 presented with abnormal sitting behaviors, in which he would keep one or both feet from touching the cage bottom when at rest (clinical score: 2.5). Neurological exams revealed diminished sensation in all extremities, and he failed to respond to hot or cold stimuli. M02 exhibited more minor disease symptoms (clinical score: 0.5) (Figure 1).

Though neurologic symptoms were observed in three of the four marmosets inoculated intravenously with HHV-6A, intravenous inoculation with HHV-6B did not result in clinical symptoms, similar to the SupT1 control inoculations. In mouse and marmoset models of EAE, weight loss is a surrogate marker of disease [50]. In our experiment, all marmosets were weighed several times per week, but none exhibited weight loss over the 25-week monitoring period (Figure 1, dashed lines).

### MRI in HHV-6 infected marmosets

**Figure 2:**
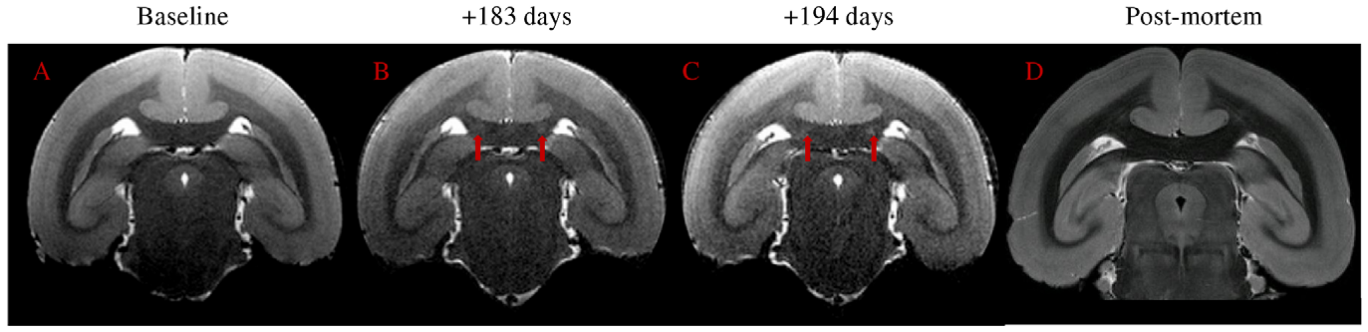
Bilateral hyperintense MRI signal in corpus callosum of M04 (red arrows), inoculated with HHV-6A intravenously. (a) Baseline, acquired before viral inoculation, (b) 183 days post-inoculation, (c) 194 days post-inoculation, (d) post-mortem scan (433 days post-inoculation).

All marmosets underwent MRI scans of the brain before viral inoculations, which served as the baseline control for each animal. Following HHV-6 inoculation, all animals were scanned monthly to assess radiologic changes from their baseline scan. As shown in Figure 2, bilateral, T2-hyperintense lesions were noted in the corpus callosum of one marmoset inoculated with HHV-6A intravenously (M04). These lesions, absent 82 days post-inoculation (Figure 2A) were noted on consecutive slices of scans conducted 173 days (Figure 2B) and 194 days (Figure 2C) post-inoculation. The lesions had resolved by the time of the post-mortem scan, which was conducted 433 days post-inoculation (Figure 2D). On day 167 post-inoculation, M04 experienced a recurrence of motor weakness in his hind limbs, corresponding to a score of 2.5 (Figure 1, solid line). This deficit was still present on days 173 and 194, when the brain abnormalities were detected. No MRI-detectable lesions were observed in the brains of the HHV-6B inoculated animals, comparable to the SupT1 vehicle controls.

### Intravenous inoculation of HHV-6A results in virus-specific IgM and IgG responses

**Figure 3:**
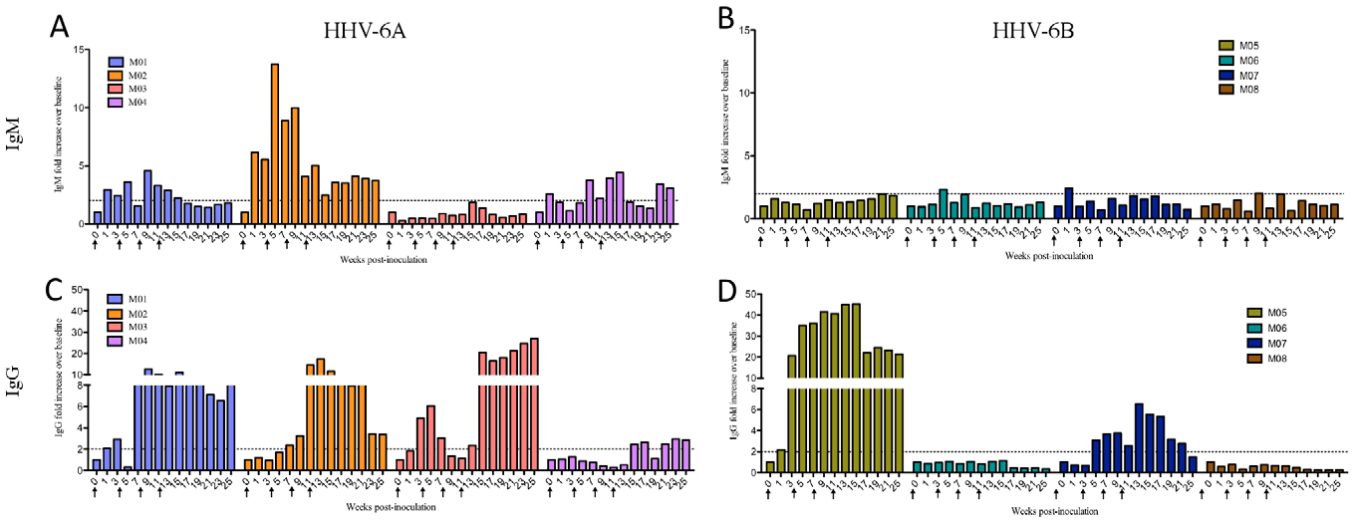
HHV-6-specific serum antibody responses of intravenously inoculated marmosets. Marmosets inoculated intravenously with HHV-6A generated greater virus-specific IgM and IgG responses than marmosets inoculated intravenously with HHV-6B. Plasma collected every two weeks was assayed for IgM and IgG reactivity to HHV-6 lysates. Results are represented as fold increases over baseline (before viral inoculation). The dotted line marks a two-fold increase above baseline, responses below which were considered negative. (A) IgM and (C) IgG responses of HHV-6A intravenously inoculated animals. (B) IgM and (D) IgG responses of HHV-6B intravenously inoculated animals.

Marmoset plasma was collected prior to HHV-6 inoculation and every two weeks post inoculation, to monitor longitudinal IgM and IgG reactivity to HHV-6 and SupT1 control lysates. Intravenous inoculation of HHV-6A led to a rapid, virus-specific IgM response that was detectable as early as week one post-inoculation (Figure 3A). Over the 25 week monitoring period, three of the four marmosets exposed to HHV-6A intravenously produced HHV-6-specific IgM responses greater than two-fold above baseline (M01, M02 and M04, Figure 3A), and all four mounted HHV-6-specific IgG responses (Figure 3C) that were detectable as early as week three post-inoculation.

**Figure 4:**
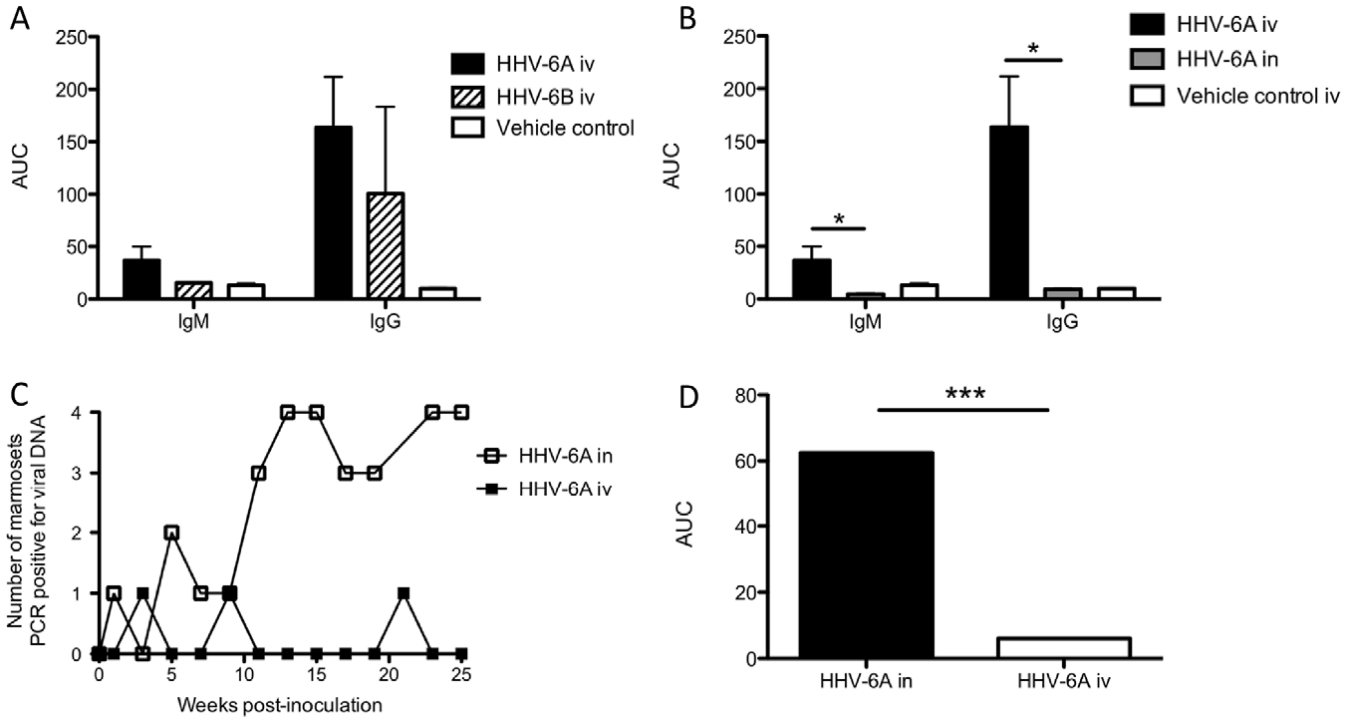
Differences in HHV-6-specific antibody responses and detection of viral DNA between experimental groups. (A) Comparison of virus-specific IgM and IgG responses between HHV-6A and HHV-6B-intravenously inoculated (iv) marmosets. AUC is calculated from Figure 3. (B) Significantly elevated virus-specific IgM and IgG responses in marmosets inoculated with HHV-6A intravenously compared to marmosets inoculated with HHV-6A intranasally (p = 0.0286, Mann Whitney U test). AUC is calculated from Figures 3, 5. (C, D) The number of marmosets testing positive for viral DNA was significantly greater in the HHV-6A intranasal group compared to the HHV-6A intravenous group (p = 0.003, Mann Whitney U test). AUC in (D) is calculated from (C). *AUC: Area under the curve.*

By contrast to what we observed in the HHV-6A intravenously inoculated marmosets, none of the marmosets inoculated intravenously with HHV-6B mounted virus-specific IgM responses (Figure 3B), and only two of the four demonstrated virus-specific IgG responses (Figure 3D). M05 mounted a robust virus-specific IgG response, the magnitude of which, in the absence of a detectable IgM response, suggests a previous exposure to this virus. M07 also mounted an IgG response, though lower in magnitude than M05 (Figure 3D). The virus-specific IgM (Figure 3B) and IgG (Figure 3D) responses of the remaining two HHV-6B intravenously inoculated marmosets, M06 and M08, increased less than two-fold over baseline, and were therefore considered negative. SupT1 control marmosets did not generate a virus-specific antibody response (data not shown). Collectively, these results demonstrate that among the intravenously inoculated marmosets, animals inoculated with HHV-6A mounted greater IgM and IgG responses compared to animals inoculated with HHV-6B, although these differences did not reach statistical significance (Figure 4A). This may be due to the small numbers of animals per group, a common limitation in NHP studies, or the heterogeneity between marmosets, which is inherent to this outbred model.

### HHV-6 DNA detection in HHV-6 intravenously inoculated marmosets

**Figure 5:**
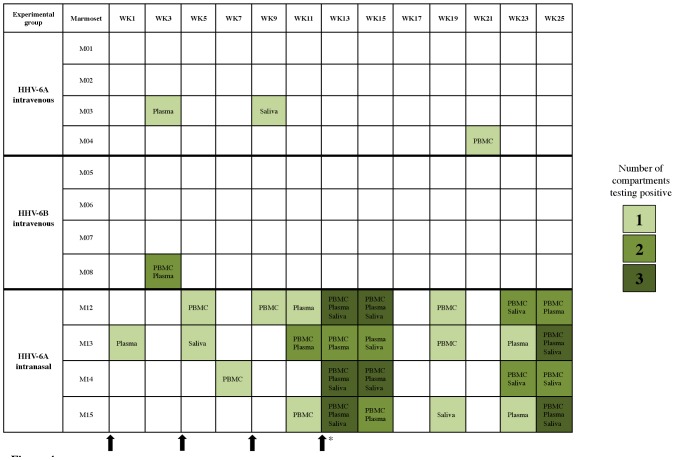
Longitudinal profiling of marmoset PBMC, plasma and saliva for HHV-6 DNA following viral inoculation. Compartments listed in shaded boxes were positive for HHV-6 DNA by nested PCR at the indicated time points post-viral inoculation. The shading intensity corresponds to the number of positive compartments. A blank box indicates that HHV-6 DNA was not detected in PBMC, plasma or saliva. Arrows indicate times of viral inoculation, and the asterisk indicates that the fourth inoculation was specific to the HHV-6A and HHV-6B intravenous groups.

In marmosets inoculated with HHV-6A or HHV-6B intravenously, viral DNA was detected infrequently in the plasma, PBMC or saliva during the 25-week monitoring period (Figure 5). Viral DNA was detected in two of the marmosets inoculated with HHV-6A, at three weeks in the plasma and nine weeks in the saliva of M03, and at 21 weeks in the PBMC of M04. Viral DNA was also detected in one marmoset inoculated with HHV-6B, at three weeks in the PBMC and plasma of M08 (Figure 5). None of the vehicle control marmosets tested positive for HHV-6 DNA (data not shown).

At euthanasia, the spleen, cervical lymph nodes (LN), olfactory bulb, heart, kidney and liver were collected and analyzed for the presence of HHV-6 DNA. Viral DNA was detected by nPCR in the tissues of two of the four euthanized HHV-6A animals, but none of the three HHV-6B euthanized animals. HHV-6 DNA was detected in the spleen of M02 and in all analyzed tissues of M03, and confirmed as HHV-6A by sequencing (data not shown).

### Absence of clinical symptoms and antibody responses when HHV-6A inoculated intranasally

As intravenous inoculation of HHV-6A but not HHV-6B led to neurologic symptoms, we similarly characterized another group of marmosets that we inoculated with HHV-6A intranasally, which represents a more physiologic route of infection. Intranasal inoculation was examined based on the recent report that HHV-6 DNA could be detected in human nasal mucus and olfactory bulb [51], suggesting the olfactory pathway as a route of transmission for this virus. As salivary glands are a known reservoir of HHV-6 and other herpesviruses [52], [53], the nasal cavity may also serve as a reservoir for HHV-6.

**Figure 6:**
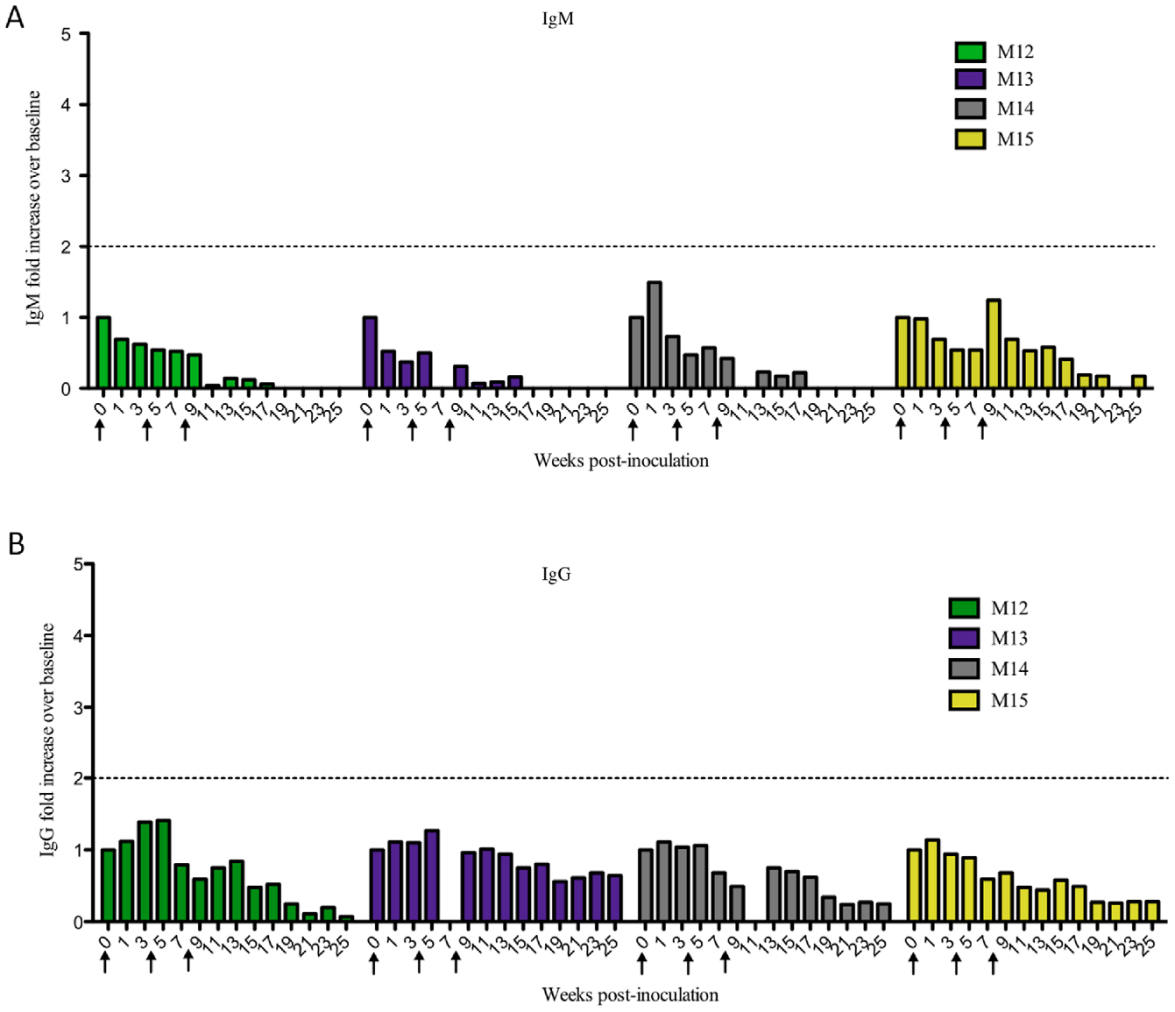
Marmosets inoculated intranasally with HHV-6A did not generate virus-specific serum IgM or IgG responses. Plasma collected every two weeks was assayed for IgM and IgG reactivity to HHV-6 lysates. Results are represented as fold increases over baseline (before viral inoculation). The dotted line marks a two-fold increase above baseline, responses below which were considered negative. (A) IgM and (B) IgG responses of HHV-6A intranasally inoculated animals.

Four naïve marmosets were inoculated with HHV-6A intranasally (Table 1) and monitored daily as previously described. During the 25-week study period, none presented with clinical signs of disease, in contrast to marmosets inoculated intravenously with this virus (Figure 1). Interestingly, unlike marmosets inoculated with HHV-6A intravenously, marmosets inoculated with HHV-6A intranasally failed to generate virus-specific IgM (Figure 6A) or IgG antibody responses (Figure 6B); all detectable serum antibodies were less than two fold above baseline throughout the 25-week study period. The HHV-6-specific IgM and IgG responses of intravenously inoculated marmosets were significantly higher compared to those of intranasally inoculated marmosets (Figure 4B).

### HHV-6A DNA detected frequently in the periphery of marmosets inoculated intranasally

Unlike marmosets inoculated with HHV-6A intravenously, marmosets inoculated with HHV-6A intranasally routinely tested positive for viral DNA in the saliva, PBMC and plasma (Figure 5). Viral DNA was detected with increasing frequency as a function of the inoculations. By week 11 (after the third and final inoculation at week 8), HHV-6A DNA was detected consistently in all marmosets, with an apparent increase during weeks 13–15 and weeks 23–25, during which viral DNA was detected in multiple compartments of most marmosets (Figure 5). The frequency of marmosets testing positive over the 25-week period was significantly elevated in the intranasally inoculated group compared to the intravenously inoculated group (Figures 4C and 4D). M15 was sacrificed for analysis as a representative of the HHV-6A intranasal group, but viral DNA was not detected in his spleen, cervical LN, olfactory bulb, heart, kidney or liver (data not shown).

### Histopathological findings

**Figure 7:**
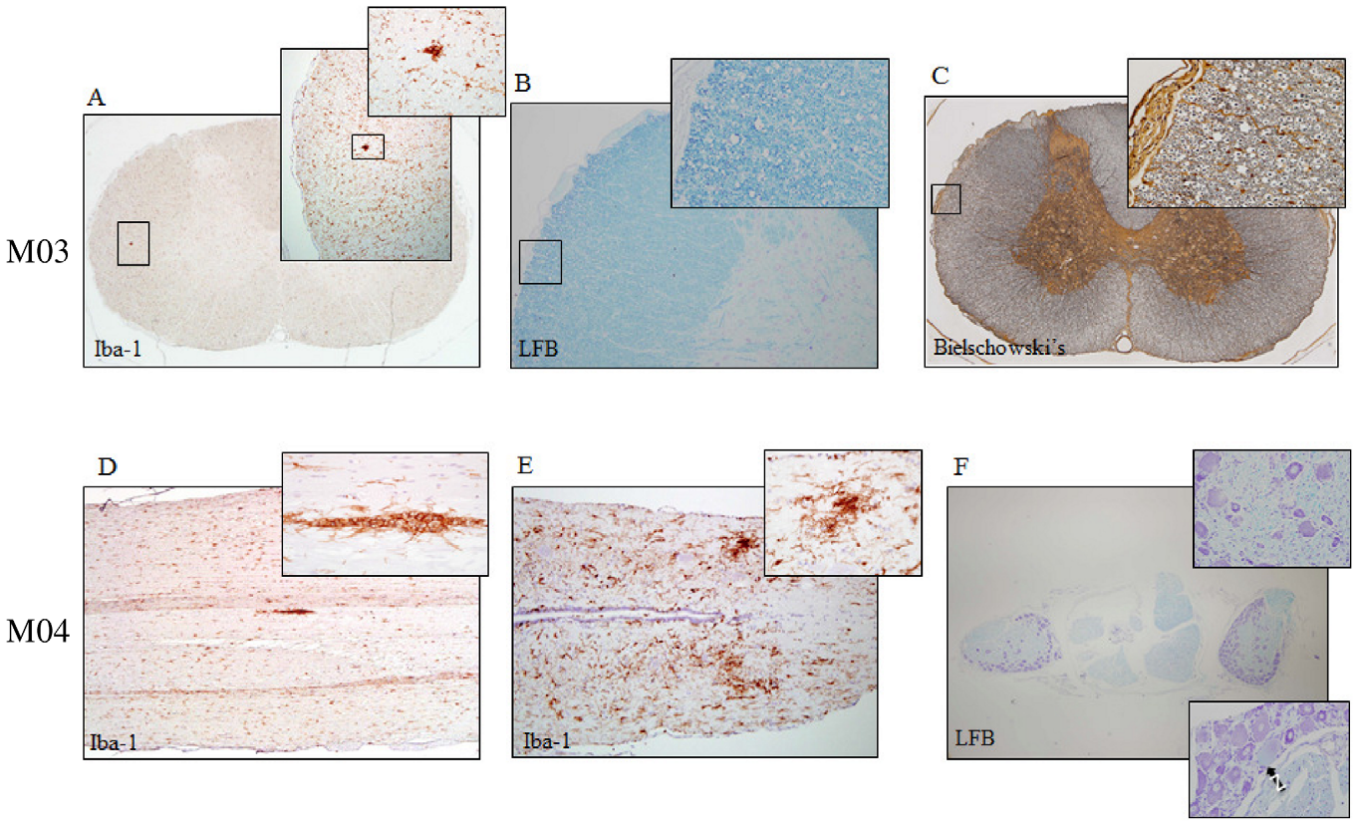
Spinal cord pathology in two HHV-6A intravenously inoculated marmosets. Iba-1 is specific for microglia and macrophages, Luxol Fast Blue (LFB) stains myelin and Bielschowski's stains neurofibrils. Cervical spinal cord pathology of M03 includes (A) microglial/macrophageal aggregates identified by Iba-1, and swollen myelin sheaths identified by (B) LFB and (C) Bielschowski's. Spinal cord pathology of M04 includes microglial/macrophageal aggregates identified by Iba-1 in the (D) thoracic and (E) lumbar spinal cord and (F) myelin abnormalities identified by LFB in the dorsal root ganglia, specifically variations in sheath size and focal neuronal chromatolysis (black arrow), indicative of mild reversible damage.

Histological evaluation of the spinal cord and brain sections showed limited pathology in M03 and M04, both of which were intravenously inoculated with HHV-6A. Iba-1 IHC showed multifocal macrophage/microglia nodules in the cervical spinal cord of M03 (Figure 7A) and mild, multifocal, gliosis in the thoracic and lumbar spinal cord of M04 (Figures 7D, 7E). Increased expression of Iba-1 is a nonspecific response to tissue injury and indicates activation of macrophages or microglia, the resident macrophages of the CNS. Macrophageal/microglial activation can be induced in the context of viral infection but is generally associated with CNS injury or disease.

LFB and Bielschowski's silver stain demonstrated mild myelin abnormalities in M03, including focal areas of swollen myelin sheaths (Figures 7B and 7C). In M04, LFB staining of the dorsal root ganglion (Figure 7F) showed variation in myelin sheath size and an abnormally high number of cells in the extracellular matrix, suggesting gliosis. The arrow in Figure 7F denotes a region of focal central neuronal chromatolysis, which is indicative of mild reversible damage. Histological staining of other study subjects did not reveal significant pathology. As euthanasia and subsequent histological examination of the tissues was performed between 136 and 433 days post-inoculation, lesions reflective of the clinical signs observed during the study may have evolved and undergone repair and healing by the time of sacrifice.

### HHV-6 DNA detected in the CNS of intravenously inoculated marmosets

Brains and spinal cords of all euthanized animals (M01–M04, M05, M06, M07, M12) were embedded in paraffin wax and sectioned, and DNA was isolated and PCR amplified for HHV-6 sequences. Of the 77 total scrolls surveyed, six (8%) were PCR positive for HHV-6 DNA (Table 1). The positive scrolls were from three of the eight marmosets sacrificed for analysis in this study, one that was inoculated with HHV-6A intravenously (M04), and two that were inoculated with HHV-6B intravenously (M05 and M07). One brain region from M04 and one from M05 were positive for viral DNA, while one spinal and three brain regions from M07 were positive. Interestingly, portions of the occipital cortex and cerebellum were positive in all three animals (Table 1). These results demonstrate that in a subset of HHV-6 intravenously inoculated marmosets, viral DNA can be detected in paraffin-embedded CNS tissue up to 14 months (in the case of M04) following intravenous viral inoculation.

## Discussion

Though highly homologous at the nucleotide level, HHV-6A and HHV-6B were recently classified as two separate viral species due to distinct biological features and disease associations (International Committee on Taxonomy of Viruses, 2011). We investigated the outcome of unique HHV-6A and HHV-6B infections in common marmosets, which are especially appropriate for the study of a virus implicated in neurologic conditions due to their natural susceptibility to infection [36], genetic and immunologic proximity to humans, and suitability for MRI monitoring [39]. Despite the association of HHV-6A with neurologic disease, the time of exposure, route of infection and related symptoms are largely unknown, partly because to our knowledge, there are no serologic assays able to distinguish between HHV-6A and HHV-6B. This marmoset model of infection therefore represents a unique opportunity to study the biology of HHV-6A infection and the mechanisms underlying its associations with the CNS.

As the human herpesviruses 6A and 6B are implicated in several CNS diseases, a non-human primate model of these viruses can be utilized to investigate how this virus may contribute to disease, and to test anti-viral therapies. For example, to date, there are no antivirals specific for HHV-6 despite widespread consensus that it is a clinically relevant pathogen. Yalcin et al. [54] followed cynomologus and African green monkeys for 33 days following subcutaneous or intravenous inoculation of HHV-6B. By contrast, we followed marmosets for more than 180 days following intravenous inoculations of HHV-6A and HHV-6B and intranasal inoculations of HHV-6A. As small non-human primates, marmosets present unique practical advantages compared to larger non-human primates such as cynomologus and African greens. Similar to Yalcin et al., we did not observe fever in any of the marmosets following the HHV-6 inoculations. Yalcin et al. observed a rash on the trunk of one African green monkey following subcutaneous inoculation with HHV-6B. We did not observe rashes in any of the marmosets inoculated with HHV-6B. These different observations may be attributed biological differences between the non-human primate species or differences between the routes of viral inoculation performed in each study.

*In vitro*, both HHV-6A and HHV-6B can infect a range of CNS cells, such as astrocytes [55], [56], oligodendrocytes [57], [58] and neural stem cells [59]. However, HHV-6A replicates and produces cytopathic effects with a greater efficiency in these cell types compared to HHV-6B. If such *in vitro* results reflect the pattern of HHV-6 infection *in vivo*, then HHV-6A may be the more involved species in CNS infections [46], [58]. Interestingly, there are data supporting the prevalence of HHV-6A in healthy CNS, which suggests that this virus may be a commensal of the brain in some instances. In a survey of healthy brains from autopsy cases, Cuomo et al. reported 32% positivity for HHV-6 DNA, with HHV-6A found three times more frequently than HHV-6B [16]. In a study of the *ex vivo* lymphoproliferative responses of MS patients, Soldan et al. reported a high percentage of MS patients (relative to healthy controls) with responses to HHV-6A, and a comparable number of patients and controls with responses to HHV-6B [24], which suggests a more prominent role for HHV-6A in this inflammatory disease of the CNS.

The results of this study lend further support to the hypothesized enhanced neuropathogenicity of HHV-6A compared to HHV-6B. In the marmoset model described here, clear neurologic symptoms were evident in three of four HHV-6A intravenously inoculated animals, characterized by gait disturbance (M04), sensory loss (M03) and incoordination (M01). By contrast, none of the HHV-6B inoculated animals exhibited symptoms. Furthermore, brain abnormalities by MRI were noted in the corpus callosum of one HHV-6A inoculated marmoset (M04), approximately two weeks following his second onset of unilateral hind limb weakness. As callosal projection neurons are considered important for motor coordination [60], MRI-detectable abnormalities in this region may be consistent with the observed unilateral motor deficit observed in M04.

Areas of abnormal CNS pathology were detected in two of the marmosets that exhibited neurologic symptoms, M03 and M04, mainly characterized by microgliosis (see Figure 7). Microglial activation is suggestive of a viral etiology but can also be associated with other causes. Following IHC analyses of the paraffin-embedded CNS tissue, adjacent sections were analyzed for the presence of viral DNA. In a subset of intravenously inoculated animals, we detected a low frequency of HHV-6 DNA in CNS tissue, demonstrating that viral DNA is present in the brain and spinal cord up to 14 months following intravenous exposure. This is consistent with reports from human studies, in which a low frequency of HHV-6 DNA (26%) was amplified from paraffin-embedded healthy control brain tissues, suggesting that HHV-6 is able to gain access to and may become a commensal pathogen of the CNS [28]. In this study, although the presence of detectable neuropathology and viral DNA in CNS tissue did not appear to correlate with clinical symptoms, this may be due to the length of time between the observed symptoms and sacrifice (up to 14 months). Additionally, the peripheral nervous system may have been affected, and its involvement was not detected during the CNS analyses performed in this study. We believe there may be additional pathology and detectable viral DNA if the animals are sacrificed closer in time to their exhibited clinical symptoms, which is under consideration for future studies.

As neurologic symptoms were observed only in the HHV-6A intravenously inoculated group, and as we had sufficient animals for one additional group of four, we chose to inoculate with HHV-6A by a more physiologic route, intranasally. Recent work from our laboratory demonstrated that HHV-6 infection may commonly occur intranasally, and that the olfactory pathway may mediate viral entry into the CNS. We demonstrated a high frequency of subjects with HHV-6 DNA in nasal mucus [51], comparable to that found in saliva, which is considered an *in vivo* reservoir of HHV-6 [52], [53]. Additionally, in a survey of brain regions from several autopsy cases, the olfactory bulb was among those from which HHV-6 DNA was detected most often [51]. Moreover, a number of neurotropic viruses are known to infect the CNS by transmission through the olfactory pathway [61]. Thus, intranasal inoculation of HHV-6 likely represents a common mode of natural infection in humans.

While anti-viral serum IgM and IgG responses were observed in marmosets inoculated with HHV-6A intravenously, marmosets inoculated with HHV-6A intranasally did not mount serum IgM or IgG responses for the 25-week study duration. This result is consistent with a report by Chang, et al. that an anti-KSHV antibody response was more elevated and sustained in marmosets infected intravenously with KSHV compared to marmosets infected orally [43]. HHV-6 DNA was detected infrequently in the PBMC, plasma or saliva of marmosets inoculated intravenously, while it was detected significantly more frequently in marmosets inoculated intranasally. This inverse correlation between antibody production and circulation of viral DNA in the periphery suggests that the absence of a specific host humoral immune response results in failure to clear the virus. These observations highlight the importance of the route of infection as a determinant in establishing humoral immunity that may serve to clear the virus. The cellular immune responses of all animals in this study are currently under investigation.

While viral DNA was rarely detected in the periphery of marmosets inoculated with HHV-6A intravenously, it was detected in several tissues of two animals: M02 and M03. M03 mounted a robust anti-HHV-6 IgG response, despite a weak IgM response, and though HHV-6 DNA was detected only once each in his plasma and saliva over the 25 week monitoring period, his liver, heart, cervical LN, kidney, spleen and olfactory bulb were all PCR positive. These data suggest that HHV-6 or other herpesviruses that establish latent infections may persist *in vivo* yet remain undetected in accessible compartments (such as, in this study, saliva, PBMC and plasma). These findings are consistent with a previous study in which low levels of HHV-6 DNA in the cerebrospinal fluid (CSF) but elevated viral DNA levels in the brain of bone marrow transplant recipients were reported. Therefore, levels of viral DNA in accessible compartments such as the serum and CSF may not accurately reflect the extent of viral infection in less accessible compartments such as the brain [62].

Interestingly, marmosets that generated anti-viral antibodies cleared the virus yet developed neurologic symptoms, while marmosets that did not generate anti-viral antibodies, and failed to clear the virus, displayed no symptoms. This observation is reminiscent of human immune-mediated diseases in which the effects of an immune response to the agent may be more damaging than the effects of the agent alone, for example in persistent viral infections resulting from the constant presence of viral antigen [63]. Antiviral antibodies are implicated in immune-mediated disease, and antiviral immune responses can initiate disease, for example myelin destruction [64]. This is consistent with our observation of mild myelin abnormalities in two of the marmosets that exhibited both clinical symptoms and anti-HHV-6 antibody responses (M03 and M04).

Collectively, these observations help to define the contributions of ubiquitous herpesviruses in the development of neurologic disease in a non-human primate model. As little is known about the acquisition and host response to HHV-6A and this species of HHV-6 has comparatively greater associations with neurologic disease, this non-human primate model of infection may further our understanding about how this ubiquitous virus may trigger or potentiate disease. More broadly, this *in vivo* model of HHV-6 infection can be used for preclinical testing of interventional strategies to interfere with the virus and further elucidate its role in human neurologic disease.

### Accession numbers/ID numbers

The NCBI reference sequence NC_001664.2 was used for HHV-6A. The NCBI reference sequence NC_000898.1 was used for HHV-6B. The Entrez gene ID numbers for the genes mentioned in the text are as follows, HHV-6A U90: 1487968; HHV-6B U90: 1497087; HHV-6A U57: 1487939; HHV-6B U57: 1497059.

## References

[1] Pellett PE, Dominguez G. (2001) Chapter 80. Human Herpesviruses 6A, 6B, and 7 and Their Replication. In: Knipe DM, Howley, P.M., editor. Fields Virology. 4th edition. Philadelphia: Lippincott, Williams & Wilkins. pp. 69–2784.

[2] Salahuddin SZ, Ablashi DV, Markham PD, Josephs SF, Sturzenegger S, et al (1986) Isolation of a new virus, HBLV, in patients with lymphoproliferative disorders. Science 234: 596–601. 2876520 10.1126/science.2876520

[3] Dominguez G, Dambaugh TR, Stamey FR, Dewhurst S, Inoue N, et al (1999) Human herpesvirus 6B genome sequence: coding content and comparison with human herpesvirus 6A. J Virol 73: 8040–8052. 10482553 10.1128/jvi.73.10.8040-8052.1999 PMC112820

[4] De Bolle L, Naesens L, De Clercq E (2005) Update on human herpesvirus 6 biology, clinical features, and therapy. Clin Microbiol Rev 18: 217–245. 15653828 10.1128/CMR.18.1.217-245.2005 PMC544175

[5] De Bolle L, Van Loon J, De Clercq E, Naesens L (2005) Quantitative analysis of human herpesvirus 6 cell tropism. J Med Virol 75: 76–85. 15543581 10.1002/jmv.20240

[6] Dewhurst S, Skrincosky D, van Loon N (1997) Human herpesvirus 6. Expert Rev Mol Med 1997: 1–17. 10.1017/S146239949700001X 14585128

[7] Zerr DM, Meier AS, Selke SS, Frenkel LM, Huang ML, et al (2005) A population-based study of primary human herpesvirus 6 infection. New England Journal of Medicine 352: 768–776. 15728809 10.1056/NEJMoa042207

[8] Yamanishi K, Okuno T, Shiraki K, Takahashi M, Kondo T, et al (1988) Identification of human herpesvirus-6 as a causal agent for exanthem subitum. Lancet 1: 1065–1067. 2896909 10.1016/s0140-6736(88)91893-4

[9] Braun DK, Dominguez G, Pellett PE (1997) Human herpesvirus 6. Clinical Microbiology Reviews 10: 521–567. 9227865 10.1128/cmr.10.3.521 PMC172933

[10] Clark DA (2000) Human herpesvirus 6. Rev Med Virol 10: 155–173. 10815027 10.1002/(sici)1099-1654(200005/06)10:3<155::aid-rmv277>3.0.co;2-6

[11] Krueger GR, Ablashi DV (2003) Human herpesvirus-6: a short review of its biological behavior. Intervirology 46: 257–269. 14555846 10.1159/000073205

[12] Luppi M, Marasca R, Barozzi P, Ferrari S, Ceccherini-Nelli L, et al (1993) Three cases of human herpesvirus-6 latent infection: integration of viral genome in peripheral blood mononuclear cell DNA. J Med Virol 40: 44–52. 8099945 10.1002/jmv.1890400110

[13] Yoshikawa T, Ihira M, Suzuki K, Suga S, Iida K, et al (2000) Human herpesvirus 6 infection after living related liver transplantation. J Med Virol 62: 52–59. 10935989

[14] Shintaku M, Kaneda D, Tada K, Katano H, Sata T (2010) Human herpes virus 6 encephalomyelitis after bone marrow transplantation: report of an autopsy case. Neuropathology 30: 50–55. 19422536 10.1111/j.1440-1789.2009.01020.x

[15] Lautenschlager I, Razonable RR (2012) Human herpesvirus-6 infections in kidney, liver, lung, and heart transplantation: review. Transpl Int 25: 493–502. 22356254 10.1111/j.1432-2277.2012.01443.x

[16] Cuomo L, Trivedi P, Cardillo MR, Gagliardi FM, Vecchione A, et al (2001) Human herpesvirus 6 infection in neoplastic and normal brain tissue. J Med Virol 63: 45–51. 11130886

[17] Yao K, Crawford JR, Komaroff AL, Ablashi DV, Jacobson S (2010) Review part 2: Human herpesvirus-6 in central nervous system diseases. J Med Virol 82: 1669–1678. 20827763 10.1002/jmv.21861 PMC4758195

[18] Tavakoli NP, Nattanmai S, Hull R, Fusco H, Dzigua L, et al (2007) Detection and typing of human herpes virus 6 by molecular methods, in specimens from patients diagnosed with encephalitis/meningitis. J Clin Microbiol 45: 3972–8. 17942643 10.1128/JCM.01692-07 PMC2168559

[19] Gewurz BE, Marty FM, Baden LR, Katz JT (2008) Human herpesvirus 6 encephalitis. Current Infectious Disease Reports 10: 292–299. 18765102 10.1007/s11908-008-0048-1

[20] Yao K, Honarmand S, Espinosa A, Akhyani N, Glaser C, et al (2009) Detection of human herpesvirus-6 in cerebrospinal fluid of patients with encephalitis. Ann Neurol 65: 257–267. 19334059 10.1002/ana.21611 PMC2666109

[21] Uesugi H, Shimizu H, Maehara T, Arai N, Nakayama H (2000) Presence of human herpesvirus 6 and herpes simplex virus detected by polymerase chain reaction in surgical tissue from temporal lobe epileptic patients. Psychiatry Clin Neurosci 54: 589–593. 11043811 10.1046/j.1440-1819.2000.00758.x

[22] Theodore WH, Epstein L, Gaillard WD, Shinnar S, Wainwright MS, et al (2008) Human herpes virus 6B: a possible role in epilepsy? Epilepsia 49: 1828–1837. 18627418 10.1111/j.1528-1167.2008.01699.x PMC2694582

[23] Alvarez-Lafuente R, De Las Heras V, Bartolome M, Garcia-Montojo M, Arroyo R (2006) Human herpesvirus 6 and multiple sclerosis: a one-year follow-up study. Brain Pathol 16: 20–27. 16612979 10.1111/j.1750-3639.2006.tb00558.x PMC8095909

[24] Soldan SS, Leist TP, Juhng KN, McFarland HF, Jacobson S (2000) Increased lymphoproliferative response to human herpesvirus type 6A variant in multiple sclerosis patients. Ann Neurol 47: 306–313. 10716249

[25] Challoner PB, Smith KT, Parker JD, MacLeod DL, Coulter SN, et al (1995) Plaque-associated expression of human herpesvirus 6 in multiple sclerosis. Proc Natl Acad Sci U S A 92: 7440–7444. 7638210 10.1073/pnas.92.16.7440 PMC41355

[26] Goodman AD, Mock DJ, Powers JM, Baker JV, Blumberg BM (2003) Human herpesvirus 6 genome and antigen in acute multiple sclerosis lesions. J Infect Dis 187: 1365–1376. 12717617 10.1086/368172

[27] Opsahl ML, Kennedy PG (2005) Early and late HHV-6 gene transcripts in multiple sclerosis lesions and normal appearing white matter. Brain 128: 516–527. 15659422 10.1093/brain/awh390 PMC7109784

[28] Cermelli C, Berti R, Soldan SS, Mayne M, D'Ambrosia JM, et al (2003) High frequency of human herpesvirus 6 DNA in multiple sclerosis plaques isolated by laser microdissection. J Infect Dis 187: 1377–1387. 12717618 10.1086/368166

[29] Akhyani N, Berti R, Brennan MB, Soldan SS, Eaton JM, et al (2000) Tissue distribution and variant characterization of human herpesvirus (HHV)-6: increased prevalence of HHV-6A in patients with multiple sclerosis. J Infect Dis 182: 1321–1325. 11023456 10.1086/315893

[30] Berti R, Brennan MB, Soldan SS, Ohayon JM, Casareto L, et al (2002) Increased detection of serum HHV-6 DNA sequences during multiple sclerosis (MS) exacerbations and correlation with parameters of MS disease progression. J Neurovirol 8: 250–256. 12053279 10.1080/13550280290049615-1

[31] Khaki M, Ghazavi A, Ghasami K, Rafiei M, Payani MA, et al (2011) Evaluation of viral antibodies in Iranian multiple sclerosis patients. Neurosciences (Riyadh) 16: 224–228. 21677611

[32] Simpson S, Taylor B, Dwyer D, Taylor J, Blizzard L, et al (2011) Anti-HHV-6 IgG titer significantly predicts subsequent relapse risk in multiple sclerosis. Multiple Sclerosis Journal 18: 799–806. 22084489 10.1177/1352458511428081

[33] Soldan SS, Berti R, Salem N, Secchiero P, Flamand L, et al (1997) Association of human herpes virus 6 (HHV-6) with multiple sclerosis: increased IgM response to HHV-6 early antigen and detection of serum HHV-6 DNA [see comments]. Nat Med 3: 1394–1397. 9396611 10.1038/nm1297-1394

[34] Fotheringham J, Donati D, Akhyani N, Fogdell-Hahn A, Vortmeyer A, et al (2007) Association of human herpesvirus-6B with mesial temporal lobe epilepsy. PLoS Med 4: e180. 17535102 10.1371/journal.pmed.0040180 PMC1880851

[35] Santoro F, Kennedy PE, Locatelli G, Malnati MS, Berger EA, et al (1999) CD46 is a cellular receptor for human herpesvirus 6. Cell 99: 817–827. 10619434 10.1016/s0092-8674(00)81678-5

[36] Murakami Y, Seya T, Kurita M, Fukui A, Ueda S, et al (1998) Molecular cloning of membrane cofactor protein (MCP; CD46) on B95a cell, an Epstein-Barr virus-transformed marmoset B cell line: B95a-MCP is susceptible to infection by the CAM, but not the Nagahata strain of the measles virus. Biochem J 330 Pt 3: 1351–1359. 9494106 10.1042/bj3301351 PMC1219282

[37] Okano H, Hikishima K, Iriki A, Sasaki E (2012) The common marmoset as a novel animal model system for biomedical and neuroscience research applications. Seminars in Fetal & Neonatal Medicine 1–5. 22871417 10.1016/j.siny.2012.07.002

[38] Uccelli A, Giunti D, Capello E, Roccatagliata L, Mancardi GL (2003) EAE in the common marmoset Callithrix jacchus. Int MS J 10: 6–12. 12906764

[39] t Hart BA, Massacesi L (2009) Clinical, pathological, and immunologic aspects of the multiple sclerosis model in common marmosets (Callithrix jacchus). J Neuropathol Exp Neurol 68: 341–355. 19337065 10.1097/NEN.0b013e31819f1d24

[40] t Hart BA, Laman JD, Bauer J, Blezer E, van Kooyk Y, et al (2004) Modelling of multiple sclerosis: lessons learned in a non-human primate. Lancet Neurol 3: 588–597. 15380155 10.1016/S1474-4422(04)00879-8

[41] Massacesi L, Genain CP, Lee-Parritz D, Letvin NL, Canfield D, et al (1995) Active and passively induced experimental autoimmune encephalomyelitis in common marmosets: a new model for multiple sclerosis. Ann Neurol 37: 519–530. 7717689 10.1002/ana.410370415

[42] Provost PJ, Keller PM, Banker FS, Keech BJ, Klein HJ, et al (1987) Successful infection of the common marmoset (Callithrix jacchus) with human varicella-zoster virus. J Virol 61: 2951–2955. 3041014 10.1128/jvi.61.10.2951-2955.1987 PMC255866

[43] Chang H, Wachtman LM, Pearson CB, Lee JS, Lee HR, et al (2009) Non-human primate model of Kaposi's sarcoma-associated herpesvirus infection. PLoS Pathog 5: e1000606. 19798430 10.1371/journal.ppat.1000606 PMC2745662

[44] Omatsu T, Moi ML, Hirayama T, Takasaki T, Nakamura S, et al (2011) Common marmoset (Callithrix jacchus) as a primate model of dengue virus infection: development of high levels of viraemia and demonstration of protective immunity. J Gen Virol 92: 2272–2280. 21697346 10.1099/vir.0.031229-0

[45] Harberts E, Yao K, Wohler JE, Maric D, Ohayon J, et al (2011) Human herpesvirus-6 entry into the central nervous system through the olfactory pathway. Proc Natl Acad Sci U S A 108: 13734–13739. 21825120 10.1073/pnas.1105143108 PMC3158203

[46] Donati D, Martinelli E, Cassiani-Ingoni R, Ahlqvist J, Hou J, et al (2005) Variant-specific tropism of human herpesvirus 6 in human astrocytes. J Virol 79: 9439–9448. 16014907 10.1128/JVI.79.15.9439-9448.2005 PMC1181567

[47] Nitsche A, Muller CW, Radonic A, Landt O, Ellerbrok H, et al (2001) Human herpesvirus 6A DNA Is detected frequently in plasma but rarely in peripheral blood leukocytes of patients after bone marrow transplantation. J Infect Dis 183: 130–133. 11076708 10.1086/317651

[48] Kap YS, Smith P, Jagessar SA, Remarque E, Blezer E, et al (2008) Fast progression of recombinant human myelin/oligodendrocyte glycoprotein (MOG)-induced experimental autoimmune encephalomyelitis in marmosets is associated with the activation of MOG34-56-specific cytotoxic T cells. J Immunol 180: 1326–1337. 18209026 10.4049/jimmunol.180.3.1326

[49] Yao K, Gagnon S, Akhyani N, Williams E, Fotheringham J, et al (2008) Reactivation of human herpesvirus-6 in natalizumab treated multiple sclerosis patients. PLoS ONE 3: e2028. 18446218 10.1371/journal.pone.0002028 PMC2323568

[50] Kap YS, Jagessar SA, van Driel N, Blezer E, Bauer J, et al (2011) Effects of early IL-17A neutralization on disease induction in a primate model of experimental autoimmune encephalomyelitis. J Neuroimmune Pharmacol 6: 341–353. 20700661 10.1007/s11481-010-9238-3 PMC3128270

[51] Harberts E, Yao K, Wohler J, Maric D, Ohayon J, et al (2011) Human herpesvirus-6 entry into the central nervous system through the olfactory pathway. PNAS 108: 13734–13739. 21825120 10.1073/pnas.1105143108 PMC3158203

[52] Levy JA, Ferro F, Greenspan D, Lennette ET (1990) Frequent isolation of HHV-6 from saliva and high seroprevalence of the virus in the population. Lancet 335: 1047–1050. 1970369 10.1016/0140-6736(90)92628-u

[53] Chen T, Hudnall SD (2006) Anatomical mapping of human herpesvirus reservoirs of infection. Mod Pathol 19: 726–737. 16528368 10.1038/modpathol.3800584

[54] Yalcin S, Mukai T, Kondo K, Ami Y, Okawa T, et al (1992) Experimental infection of cynomolgus and African green monkeys with human herpesvirus 6. J Gen Virol 73 Pt 7: 1673–1677. 1321207 10.1099/0022-1317-73-7-1673

[55] He J, McCarthy M, Zhou Y, Chandran B, Wood C (1996) Infection of primary human fetal astrocytes by human herpesvirus 6. J Virol 70: 1296–1300. 8551599 10.1128/jvi.70.2.1296-1300.1996 PMC189947

[56] Ahlqvist J, Donati D, Martinelli E, Akhyani N, Hou J, et al (2006) Complete replication cycle and acquisition of tegument in nucleus of human herpesvirus 6A in astrocytes and in T-cells. J Med Virol 78: 1542–1553. 17063514 10.1002/jmv.20737

[57] Albright AV, Lavi E, Black JB, Goldberg S, O'Connor MJ, et al (1998) The effect of human herpesvirus-6 (HHV-6) on cultured human neural cells: oligodendrocytes and microglia. J Neurovirol 4: 486–494. 9839646 10.3109/13550289809113493

[58] Ahlqvist J, Fotheringham J, Akhyani N, Yao K, Fogdell-Hahn A, et al (2005) Differential tropism of human herpesvirus 6 (HHV-6) variants and induction of latency by HHV-6A in oligodendrocytes. J Neurovirol 11: 384–394. 16162481 10.1080/13550280591002379 PMC7095087

[59] De Filippis L, Foglieni C, Silva S, Vescovi AL, Lusso P, et al (2006) Differentiated human neural stem cells: a new ex vivo model to study HHV-6 infection of the central nervous system. J Clin Virol 37 Suppl 1: S27–32. 17276364 10.1016/S1386-6532(06)70008-7

[60] Rouiller EM, Babalian A, Kazennikov O, Moret V, Yu XH, et al (1994) Transcallosal connections of the distal forelimb representations of the primary and supplementary motor cortical areas in macaque monkeys. Exp Brain Res 102: 227–243. 7705502 10.1007/BF00227511

[61] Mori I, Nishiyama Y, Yokochi T, Kimura Y (2005) Olfactory transmission of neurotropic viruses. J Neurovirol 11: 129–137. 16036791 10.1080/13550280590922793

[62] Fotheringham J, Akhyani N, Vortmeyer A, Donati D, Williams E, et al (2007) Detection of active human herpesvirus-6 infection in the brain: correlation with polymerase chain reaction detection in cerebrospinal fluid. J Infect Dis 195: 450–454. 17205485 10.1086/510757

[63] Fujinami RS, von Herrath MG, Christen U, Whitton JL (2006) Molecular mimicry, bystander activation, or viral persistence: infections and autoimmune disease. Clin Microbiol Rev 19: 80–94. 16418524 10.1128/CMR.19.1.80-94.2006 PMC1360274

[64] Yamada M, Zurbriggen A, Fujinami RS (1990) Monoclonal antibody to Theiler's murine encephalomyelitis virus defines a determinant on myelin and oligodendrocytes, and augments demyelination in experimental allergic encephalomyelitis. J Exp Med 171: 1893–1907. 1693653 10.1084/jem.171.6.1893 PMC2187947

